# Supplementary material for: Phasic modulation of visual representations during sustained attention
Source: Eur J Neurosci. 2021 Jan 6;55(11-12):3191–208. doi: 10.1111/ejn.15084 (PMC9543919; doi:10.1111/ejn.15084)
Supplement: Supplementary file 1 — Supplementary Material [file EJN-55-3191-s001.docx]

European Journal of Neuroscience

Rhythms in Cognition: Revisiting the Evidence

Research report

Title: Phasic modulation of visual representations during sustained attention

Author names: Mats W.J. van Es, Tom R. Marshall, Eelke Spaak, Ole Jensen, Jan-Mathijs Schoffelen

**Supporting information**

Table 1. Single subject data from the control analysis on decoding on eye-tracker data. The most discriminative trials were removed from the eye-tracker data until accuracy was at chance level. The same trials were removed from the MEG data and performance with and without removal were compared. The columns in the ‘eye-tracker decoding’-field indicate average accuracy over 10 folds, before and after removal of the selected trials, and the p-value from a T-test of the accuracy relative to the accuracy resulting from shuffled class-labels. The columns in the ‘MEG decoding’-field indicate the accuracy before and after removal of the selected pseudo-observations, and the p-value is the result from a T-test, comparing the accuracy before and after removal of the selected pseudo-observations over folds (i.e. a p-value of >0.05 indicates no difference in MEG decoding accuracy despite removing the pseudo-observations that were furthest away from the decision boundary in the eye-tracking decoding). The last column from in both parts indicate the percentage of pseudo-observations that were removed from the data. Cells are empty in case performance based on eye-tracking data was already at chance level. acc: accuracy; pval: p-value.

|  | attend-left trials | | | | | | | | attend-right trials | | | | | | | |
| --- | --- | --- | --- | --- | --- | --- | --- | --- | --- | --- | --- | --- | --- | --- | --- | --- |
|  | eye-tracker decoding | | | | MEG decoding | | | % removed pseudo-observations | eye-tracker decoding | | | | MEG decoding | | | % removed pseudo-observations |
| subj | pre | | post | | acc (%) | | pval |  | pre | | post | | acc (%) | | pval |  |
|  | acc (%) | pval | acc (%) | pval | pre | post |  |  | acc (%) | pval | acc (%) | pval | pre | post |  |  |
| 1 | 54.5 | 0.00040 | 50.3 | 0.84 | 70.4 | 70.1 | 0.70 | 6.0 | 51.8 | 0.038 | 50.6 | 0.32 | 71.5 | 71.5 | 0.98 | 1.5 |
| 2 | 56.0 | 0.0053 | 51.5 | 0.16 | 70.9 | 71.0 | 0.89 | 8.3 | 50.8 | 0.087 | - | - | 71.2 | 71.5 | - | 0.0 |
| 3 | 52.6 | 0.0018 | 51.4 | 0.40 | 69.4 | 69.4 | 0.98 | 2.3 | 51.6 | 0.038 | 50.7 | 0.53 | 67.8 | 68.2 | 0.61 | 0.73 |
| 4 | 55.1 | 0.0037 | 49.8 | 0.40 | 72.9 | 72.9 | 0.93 | 6.3 | 50.1 | 0.78 | - | - | 71.5 | 71.5 | - | 0.0 |
| 5 | 52.8 | 0.0048 | 50.8 | 0.68 | 68.2 | 68.0 | 0.56 | 3.0 | 52.1 | 0.016 | 50.5 | 0.12 | 69.9 | 70.1 | 0.78 | 1.3 |
| 6 | 51.8 | 0.023 | 51.3 | 0.12 | 71.2 | 71.2 | 0.93 | 0.92 | 55.1 | 0.0014 | 50.6 | 0.85 | 72.8 | 73.3 | 0.34 | 5.9 |
| 7 | 50.2 | 0.61 | - | - | 65.9 | 66.1 | - | 0.0 | 53.1 | 0.00020 | 49.9 | 0.89 | 66.2 | 66.0 | 0.76 | 5.7 |
| 8 | 54.1 | 0.0038 | 49.9 | 0.79 | 69.1 | 68.9 | 0.49 | 5.0 | 52.1 | 0.048 | 51.2 | 0.42 | 69.0 | 68.7 | 0.64 | 2.3 |
| 9 | 53.3 | 0.0029 | 50.2 | 0.39 | 72.0 | 71.9 | 0.89 | 4.1 | 52.2 | 0.014 | 51.4 | 0.17 | 70.4 | 70.5 | 0.85 | 2.0 |
| 10 | 55.6 | 0.0017 | 50.6 | 0.29 | 70.1 | 69.9 | 0.81 | 8.3 | 51.7 | 0.0071 | 51.0 | 0.91 | 69.9 | 69.8 | 0.82 | 1.4 |


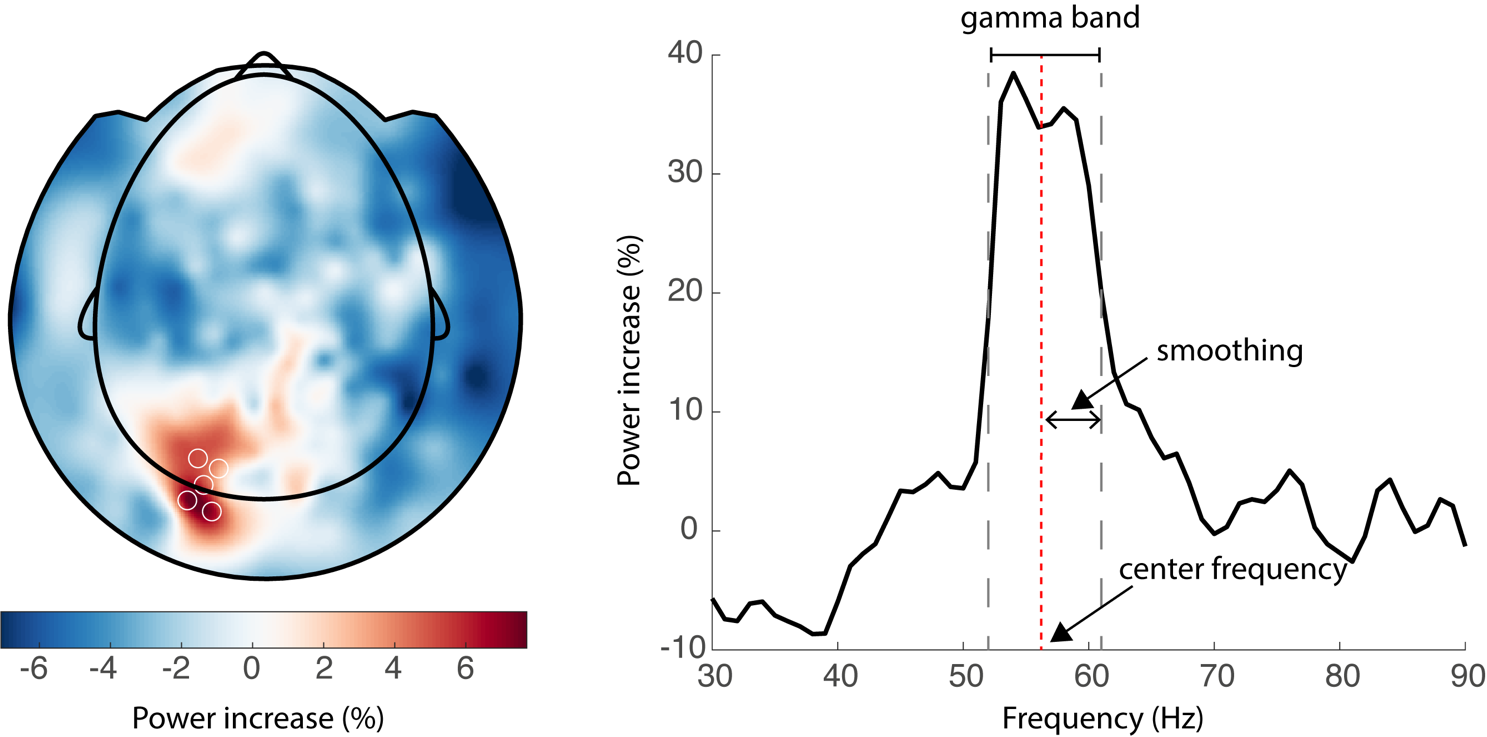


Figure S1. The subject specific gamma for a representative subject. The power spectrum of the MEG sensors with the largest gamma band increase (white circles in topography on the left) were averaged. From the power average (right) shows the gamma band and center frequency.


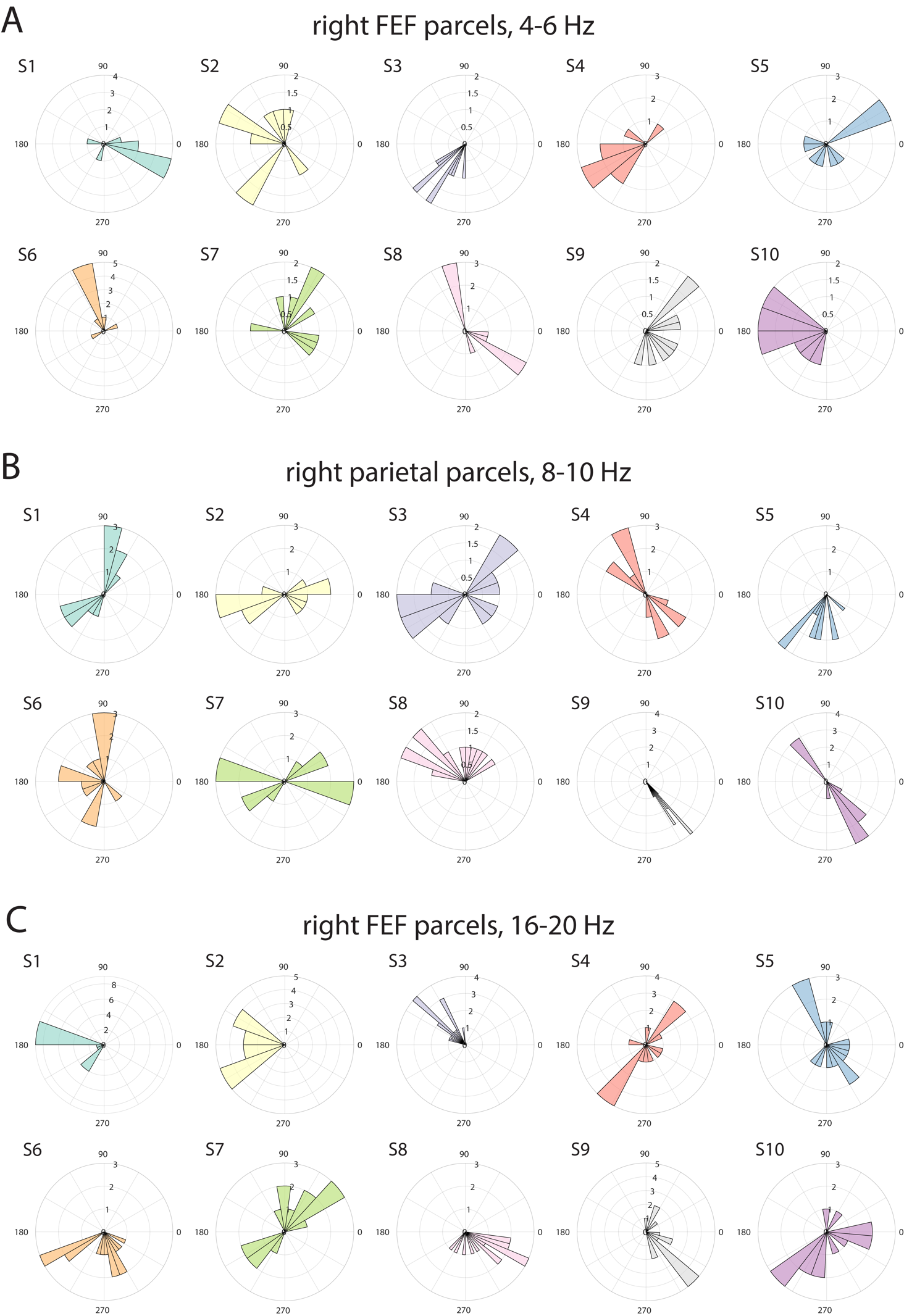


Figure S2. Optimal phase for decoding in attend-left trials. Histograms of optimal phases over parcels and frequencies that contain the strongest phasic modulation effect. Each subplot corresponds to the subplot in figure 7A-C in the same order and subject’s colors are matched with the right most panel in those subplots. Note that phase polarities are ambiguous (see main text): bimodal distributions with a 180 degree difference suggest similar optimal phases of frequencies and parcels.


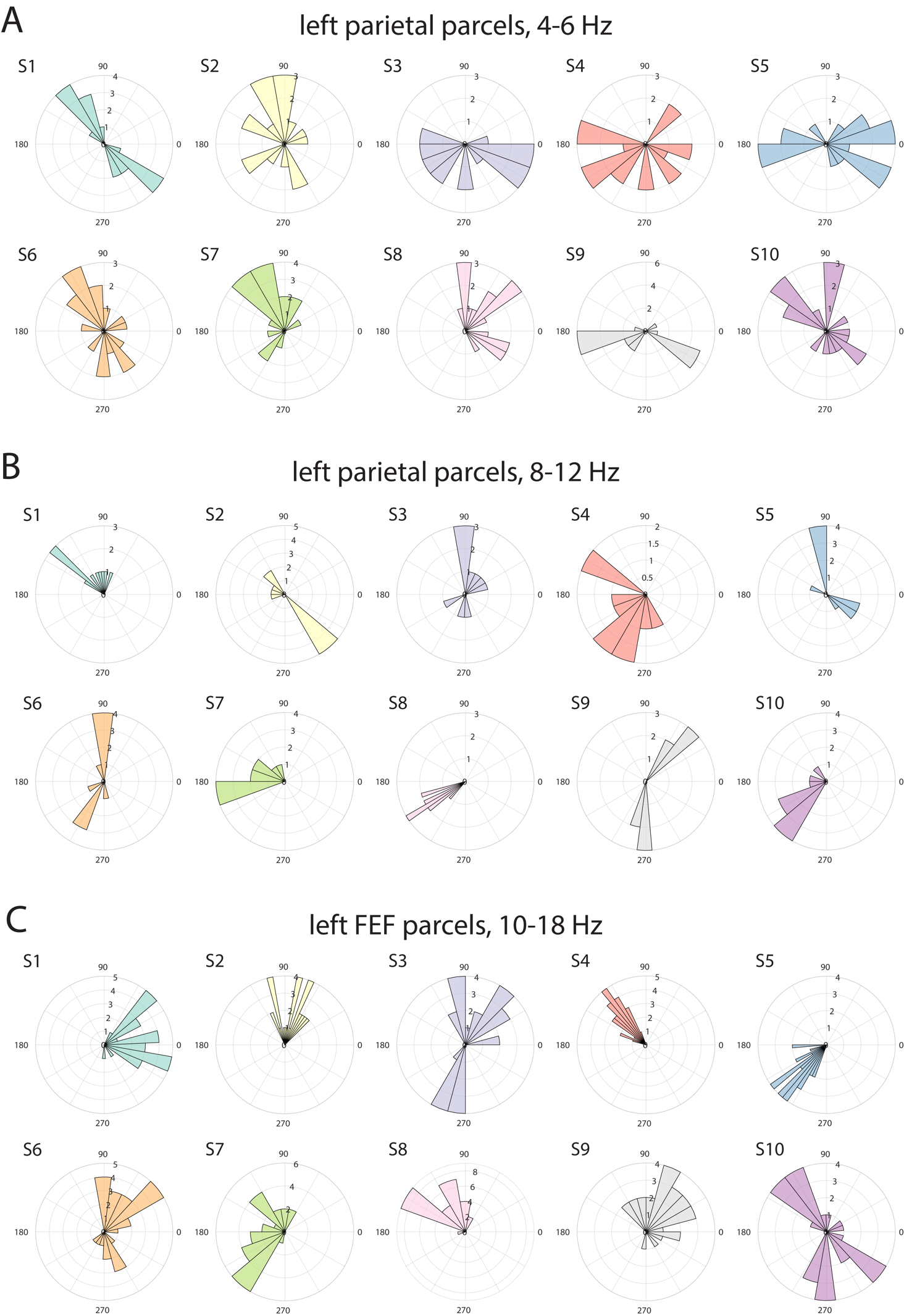


Figure S3. Optimal phase for decoding in attend-right trials. Histograms of optimal phases over parcels and frequencies that contain the strongest phasic modulation effect. Each subplot corresponds to the subplot in figure 7D-F in the same order, and subject’s colors are matched with the right most panel those subplots. Note that phase polarities are ambiguous (see main text): bimodal distributions with a 180 degree difference suggest similar optimal phases of frequencies and parcels.
